# 

**Published:** 1886-08

**Authors:** 


### TO SUBSCRIBERS.

Bills for such as are indebted to this journal will be enclosed in this and the succeeding numbers. We bespeak for them prompt attention.

